# Association of Circadian Clock Gene Expression with Glioma Tumor Microenvironment and Patient Survival

**DOI:** 10.3390/cancers13112756

**Published:** 2021-06-02

**Authors:** Julianie De La Cruz Minyety, Dorela D. Shuboni-Mulligan, Nicole Briceno, Demarrius Young, Mark R. Gilbert, Orieta Celiku, Terri S. Armstrong

**Affiliations:** Neuro-Oncology Branch, National Cancer Institute, National Institutes of Health, Bethesda, MD 20814, USA; delacruzminyej2@mail.nih.gov (J.D.L.C.M.); nicole.briceno@nih.gov (N.B.); youngdeml@mail.nih.gov (D.Y.J.); mark.gilbert@nih.gov (M.R.G.); terri.armstrong@nih.gov (T.S.A.)

**Keywords:** circadian clock genes, *PER2*, glioma, *IDH* mutational status, immune signature

## Abstract

**Simple Summary:**

Gliomas are the most common type of malignant primary brain tumors and are classified according to the cell of origin and genetic features, which can help predict the prognosis and treatment sensitivity. Improving the prognosis remains a challenge; however, chronobiology is a promising field for future works, as circadian clock genes are linked to the tumor biology and outcomes in multiple cancers, including glioma. Here, we examined the relationship of circadian clock genes, *IDH* mutational status, and prognosis in glioma patients by using unsupervised clustering of the expression of 13 clock genes. We further explored the expression of the clock genes across the tumor regions and cell subpopulations, highlighting the importance of the tumor microenvironment in researching circadian rhythms in cancer. Our research is important for understanding how best to target circadian rhythms to improve patient outcomes in neuro-oncology.

**Abstract:**

Circadian clock genes have been linked to clinical outcomes in cancer, including gliomas. However, these studies have not accounted for established markers that predict the prognosis, including mutations in Isocitrate Dehydrogenase (*IDH*), which characterize the majority of lower-grade gliomas and secondary high-grade gliomas. To demonstrate the connection between circadian clock genes and glioma outcomes while accounting for the *IDH* mutational status, we analyzed multiple publicly available gene expression datasets. The unsupervised clustering of 13 clock gene transcriptomic signatures from The Cancer Genome Atlas showed distinct molecular subtypes representing different disease states and showed the differential prognosis of these groups by a Kaplan–Meier analysis. Further analyses of these groups showed that a low period (*PER*) gene expression was associated with the negative prognosis and enrichment of the immune signaling pathways. These findings prompted the exploration of the relationship between the microenvironment and clock genes in additional datasets. Circadian clock gene expression was found to be differentially expressed across the anatomical tumor location and cell type. Thus, the circadian clock expression is a potential predictive biomarker in glioma, and further mechanistic studies to elucidate the connections between the circadian clock and microenvironment are warranted.

## 1. Introduction

Gliomas are the most common malignancy of central nervous system origin, whose broad classification includes both astrocytomas (which include glioblastomas) and oligodendrogliomas [1,2]. Despite advances in clinical research, the prognosis for these diseases remains dismal, with the survival of patients with low-grade gliomas (LGG) (grades 2 and 3) averaging 5–10 years [3] and the survival of patients with high-grade gliomas (grades 4) averaging 1 to 2 years [4,5]. Multiple markers have been identified as critical for predicting the prognosis and treatment sensitivity, such as Isocitrate Dehydrogenase 1/2 (*IDH*) mutational status, *MGMT* promoter methylation status, codeletion of chromosome arms 1p and 19q, and *EGFR* amplification [6,7,8,9,10]. *IDH* mutations, in particular, confer a better prognosis across the glioma grades. For example, patients with *IDH*-mutated grade 4 astrocytoma have a median survival of 31 months, whereas patients with grade 4 *IDH* wild-type astrocytomas (glioblastomas) only 15 months [11]. These survival differences reflect fundamental differences in tumor biology that are directly related to the presence or absence of an *IDH* mutation. IDH is a key metabolic enzyme of the tricarboxylic acid (TCA) cycle, where it catalyzes the conversion of isocitrate into alpha-ketoglutarate (alpha-KG) [12]. The *IDH* mutations encountered in gliomas result in both an impairment of the ability of IDH to perform this conversion and acquisition of a novel catalytic function that enables IDH to produce 2-hydroxyglutarate (2-HG) from alpha-KG [13,14]. The accumulation of 2-HG caused by the *IDH* mutations alters the cellular processes responsible for gliomagenesis, including DNA methylation, gene expression levels, stem cell differentiation, cellular redox states, and DNA repair [11,12,13,14,15,16]. The dampening of the wild-type metabolic function in mutant IDH cells leads to the formation of tumors that are often initially indolent and not reliant on aerobic glycolysis (non-Warburg-like) [17]. However, many lower-grade *IDH*-mutated astrocytomas will eventually transform into high-grade gliomas that have many of the morphologic characteristics of glioblastoma (*IDH* wild-type WHO Grade 4), such as vascular proliferation and necrosis, and typically exhibit an alteration of the metabolism from the neomorphic mutant IDH-driven metabolic program to the Warburg-like or aerobic glycolysis [18].

Many of the cellular processes responsible for gliomagenesis and progression are also regulated by circadian clock genes and, therefore, have daily rhythms (24-h cycles) of expression in healthy tissues [19,20,21,22]. Circadian rhythms modulate cellular functions via a transcriptional-translational feedback loop (TTFL) comprised of core clock genes: transcriptional activators *CLOCK*, and *ARNTL* and transcriptional repressors *PER1/2/3* and *CRY1/2* [23]. Within the central pacemaker of the brain, the suprachiasmatic nucleus (SCN), the *PER* gene expression is synchronized and stimulated by the onset of light. PER and CRY proteins, once transcribed, are translocated into the nucleus to interact with CLOCK and ARNTL, which then suppress the expression of PER and CRY [24]. Additional TTFLs complement the core clock components, including RORα/β/γ, REV-ERBα/β, DBP, TEF, and HLF [25]. These feedback loops impact the transcription of *ARNTL* and ensure an antiphase oscillation of the gene relative to PER and CRY [26]. Together, all the TTFLs generate rhythmic fluctuations in the transcriptome and modulate cellular processes via the regulation of clock-controlled genes (CCGs). Expression of these clock genes and regulation of CCGs are dependent on the tissue and cell types examined [27,28]. Across multiple cancers, disruption of the biological clock by environmental factors or through mutations in the circadian pathway can lead to an increased risk of tumorigenesis [29]. Differential expression of the core clock genes in cancer leads to alterations in the activation and/or inhibition of important oncogenic and tumor suppressive pathways [30]. Our laboratory reported a relationship between polymorphisms in *ARNTL* and *PER2* and treatment effects in patients with brain tumors [31]. Most of these studies examined the impact of clock genes on bulk samples of tumors. However, gliomas are highly heterogeneous and display a mixture of cell subpopulations with distinct transcriptomic profiles, raising concerns that these prior results may not provide a complete picture of clock gene effects on tumor biology [32,33]. Additionally, given the infiltrative nature and prominent vascular proliferation in glioblastoma, noncancer cells in the microenvironment may further complicate the analysis. A better understanding of how circadian clock genes may impact all components of the tumors across the glioma landscape will be key to translating the findings into the clinical setting.

In recent literature, the core clock gene expression level was found to be an effective predictor of overall survival in glioma patients [30,34]. These studies, however, did not account for the *IDH* mutational status of tissue or cell samples when studying the effects of core clock genes in glioma. In this study, we assessed the relationship of circadian pathway genes, *IDH* mutational status, and prognosis in glioma patients by analyzing the molecular profiling and clinical data from The Cancer Genome Atlas (TCGA) [35] and published data from Gao and colleagues [36]. Additionally, we explored questions about the importance of the tumor microenvironment and cell types on the clock gene expression levels using publicly available RNA-seq datasets in (1) bulk samples from different anatomical locations within a tumor [37] and (2) cell types identified and profiled at the single-cell level within tumors [38,39]. Our results provide an analysis of the clock genes across the glioma landscape by accounting for the *IDH* mutational status while highlighting the importance of circadian rhythms in the heterogeneous microenvironments and cell types that compose glioma.

## 2. Results

### 2.1. Circadian Clock Genes Define Prognostically Relevant Transcriptomic Subtypes in Glioma

To study the transcriptomic landscape of circadian clock genes in glioma and any coordinated patterns associated with prognosis, we utilized RNA-seq data from (668 grades 2–4) glioma patient samples (428 *IDH*-mutant, 233 *IDH* wild-type, and 7 of unconfirmed *IDH* status) from The Cancer Genome Atlas (TCGA) [40,41]. We performed an unsupervised consensus clustering of the samples based on the similarity of the transcriptomic profile of the (13) clock genes, members of the Kyoto Encyclopedia Genes and Genomes (KEGG) circadian rhythm pathway [42], which allowed us to appraise the patterns in the clock gene transcriptomic profiles and their association with known glioma prognostic markers, including age, *IDH* status, and sex. We determined the optimal number of clusters by considering a split of the samples into two to seven clusters and determined that four clusters (named Circadian1–Circadian4) had an optimal relative stability of the cluster assignment and composition (according to age and *IDH* mutational status) over multiple runs of the algorithm. This resulted in two pairs of clusters, each of which was characterized by similar age and sex distributions and *IDH* mutation compositions (Table 1). Circadian1 and Circadian2 were enriched for the *IDH*-mutated samples, whereas Circadian3 and Circadian4 were enriched for the *IDH* wild-type samples. We further annotated the samples with their TCGA methylation classes (LGm1-LGm6) [7], which have been shown to have a strong correspondence with the primary central nervous system tumor methylation classes increasingly used alongside the histopathological diagnosis [43,44] (Table 1). 

**Figure 1:**
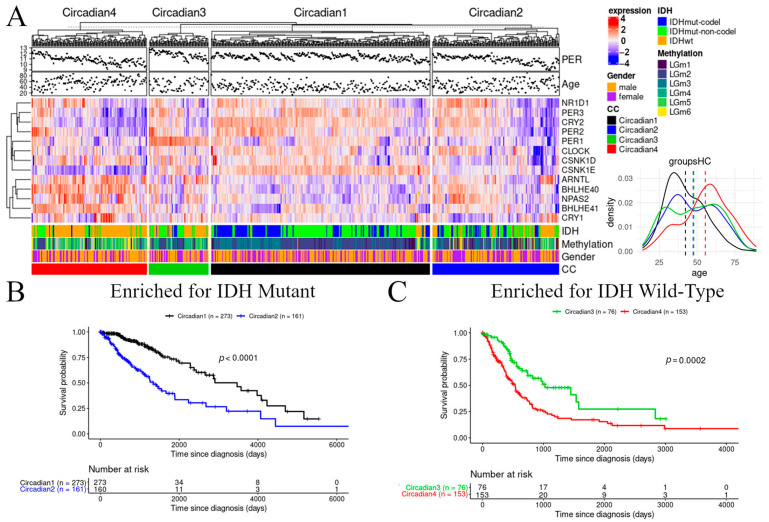
Prognostic relevance of *PER* in unsupervised clusters in the TCGA dataset. (**A**) Unsupervised clustering of patient samples identifies 4 clusters: one with enriched for *IDH* mutated samples and high *PER* expression (Circadian1, black), one with enriched for *IDH*-mutated samples and low *PER* expression (Circadian2, blue), one enriched for *IDH* wild-type samples and high *PER* expression (Circadian3, green), and one enriched for *IDH* wild-type and low *PER* expression (Circadian4, red). The heatmap shows the upregulation (red) and downregulation (blue) of clock genes for each cluster (Circadian1–4), as well as annotation bars for patient sex and TCGA methylation classes of the samples. Inlet graph demonstrates age distribution across the clusters. (**B**,**C**) Kaplan–Meier curves for clusters enriched for *IDH*-mutant (**B**) and *IDH* wild-type (**C**). Favorable prognosis was associated with a higher *PER* expression regardless of the *IDH* mutation composition.

A coordinated pattern of expression can be observed within the clusters, with *PER2, CRY2*, *NR1D1*, and *PER3* forming a core of positively correlated genes (Figure S1). *PER1/2/3*, individually, were observed to be distinctly different across the clusters, so their expression levels were averaged for each sample to obtain a “meta” *PER* gene expression value reflective of the overall *PER* expression for that sample. Clusters Circadian1 and Circadian3 were characterized by higher levels of *PER* expression compared to Circadian2 and Circadian4, respectively (Figure 1A). *BHLH40/BHLH41*, whose products can interact with ARNTL to suppress the transactivation of PER, had lower levels of expression in Circadian1 versus Circadian2 and Circadian3 versus Circadian4 (Figure S2). A further analysis compared the individual clock gene expression between the TCGA clusters (comparing Circadian2 versus Circadian1 and Circadian4 versus Circadian3; Table S1).

The transcriptomic dichotomies between clusters led to hypothesizing that these subtypes represent different disease states and, thus, analyzing whether there were differences in survival between them. The Kaplan–Meier analysis revealed pairwise differences in the overall survival across the clusters, except between Circadian2 and Circadian3 (Table S2). When comparing the clusters according to their enrichment for *IDH*-mutated or wild-type samples, we found an association between a higher expression of *PER* and better overall survival (Figure 1B,C); this association was found both for the groups enriched for *IDH*-mutated samples (Circadian1 and Circadian2) (log-rank *p* < 0.0001), as well as the groups enriched for *IDH* wild-type samples (Circadian3 and Circadian4) (*p* = 2 × 10^−4^).

To further explore whether similar transcriptomic subtypes based on clock gene expression can be observed in low-grade glioma patients, we examined an additional independent dataset comprised of gene expression profiles from (195) low-grade (2) glioma samples (166 *IDH*-mutant, and 14 *IDH* wild-type) from Gao and colleagues [36]. Using unsupervised consensus clustering, we determined the optimal number of clusters to be five (Gao CC1 to CC5, Figure S3A). Cluster Gao CC1 had the highest *PER* expression, while Gao CC3 and Gao CC4 had the lowest levels of expression. In the absence of overall survival information, we performed an analysis of the progression-free survival on these groups. An association of the progression free survival with the overall *PER* expression was found in this cohort: patients in Gao CC1 had longer progression-free survival compared to those in both Gao CC3 (*p* = 0.009, Figure S3B) and Gao CC4 (*p* = 0.027, Figure S3B).

### 2.2. Period Gene Expression Is a Biomarker of Survival in Glioma

**Figure 2:**
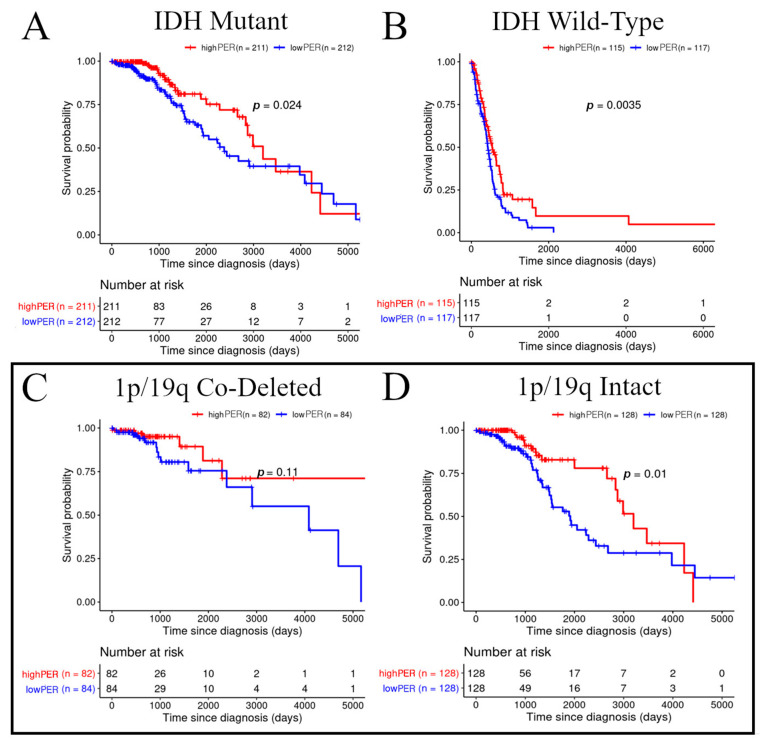
Clinical Relevance of *PER* in the TCGA dataset stratified by the *IDH* mutation and 1p/19q co-deletion status. (**A**–**D**) Kaplan–Meier curves for *IDH*-mutant (**A**), *IDH* wild-type (**B**), 1p/19q co-deleted (**C**), and 1p/19q intact (**D**) samples stratified by the median *PER* expression levels. The 1p/19q co-deleted and 1p/19q intact are subdivisions of all the *IDH*-mutant tumors. High expression (red) represents above the median of the *PER* expression, and low expression (blue) represents below the median of the *PER* expression.

The unsupervised clustering analysis demonstrated the existence of prognostic transcriptomic subtypes derived from all circadian clock genes and suggested that *PER* expression might be responsible for the survival differences. However, since the unsupervised clustering analysis resulted in groups with a mixed *IDH* mutation status, we next performed a survival analysis on the overall cohort stratified by the *IDH* status and median *PER* expression (Table 1). The above median *PER* expression was confirmed to be beneficial for both *IDH*-mutated (*p* = 0.024, Figure 2A) and *IDH* wild-type gliomas (*p* = 0.0035, Figure 2B). Within the *IDH*-mutant group, we interrogated the relationship between the 1p/19q co-deletion status and the impact of the *PER* expression levels on survival. The Kaplan–Meier analysis of cohorts stratified by the 1p/19q co-deletion status and around the median of the *PER* expression levels showed that the association of *PER* expression with overall survival was statistically significant for tumors with intact 1p/19q (*p* = 0.01) (astrocytic lineage) (Figure 2C,D) but did not achieve statistical significance in the 1p/19q co-deleted (codel) cohort (molecularly defined as oligodendroglioma). To account for age at diagnosis and sex, we performed a Cox-proportional hazards analysis that included continuous variables of *PER* expression, age at diagnosis, sex, and *IDH* mutational status. The *PER* expression was found to be an independent prognostic factor in glioma (HR = 0.73, *p* = 0.005). As anticipated from known associations, an increase in age was found to be associated with a worse prognosis (HR = 1.04, *p* < 0.001), as was the *IDH* wild-type status (HR = 5.62, *p* < 0.001) compared to the *IDH*-mutant status; sex was not found statistically significant (male versus female HR = 0.89, *p* = 0.473). These results indicate that the *PER* expression is an important predictor of survival in glioma independent of the *IDH* mutational status.

The consensus clustering analysis revealed multigene patterns characterizing some of the clusters, leading us to also assess the individual contribution of the clock genes in a multivariate Cox model of the 13 clock gene expression as continuous covariates and other key factors (age, sex, and *IDH* mutational status) (Table S3, full model). As before, an increase in age and *IDH* wild-type status were found to be associated with an increased risk of death (age HR = 1.05, *p* < 0.001; *IDH* wild-type versus mutant HR = 3.89, *p* < 0.001), but the sex was not statistically significant (male versus female HR = 0.83, *p* = 0.257). Of the circadian clock genes, *PER2* (HR = 0.66, *p* = 0.004) and *CSNK1D* (HR = 2.37, *p* = 0.005) were associated with survival; in particular, a higher *PER2* expression was associated with a decreased risk of death, whereas a higher *CSNK1D* expression was associated with an increased risk of death. These two genes’ contributions remained significant in a model that included the patient age, sex, and *IDH* mutational status (Table S3, reduced model). Interestingly, the expression of *CSNK1D* was not found to be different between Circadian4 and Circadian3 but was decreased in Circadian2 compared to Circadian1 (Table S1).

### 2.3. Low PER Expression Is Associated with Upregulation of Immune Signaling Pathways

**Figure 3:**
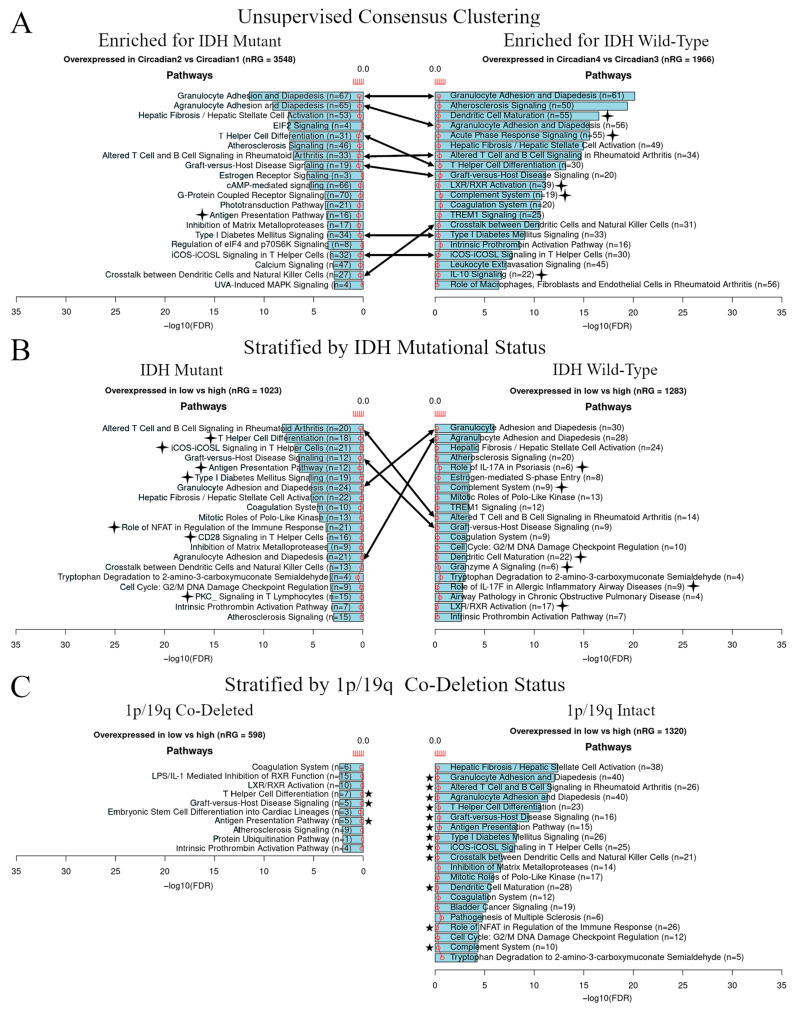
Low *PER* expression is associated with the upregulation of immune pathways. (**A**–**C**) Functional enrichment analysis showing overexpressed pathways in low *PER* versus high *PER* enriched for *IDH*-mutated and *IDH* wild-type clusters (**A**). Analysis showing overexpressed pathways in low versus high *PER* expression stratified by the median *PER* expression levels in samples stratified by *IDH* mutation (**B**) and 1p/19q co-deletion status (**C**). Lines with arrows show immune pathways commonly overexpressed across groups. Stars represent the immune pathways overexpressed uniquely in a group. The red line shows the ratio of list genes found in each pathway over the total number of genes in that pathway.

In recent publications, low *PER* expression has been postulated to lead to an increased risk of tumorigenesis for multiple cancer types [45,46,47,48]. The association between *PER* expression and prognosis in our results led us to search for potential mechanisms responsible for worsened prognosis with dropping *PER* expression levels. We performed differential gene expression analysis and functional pathway enrichment analysis between TCGA clusters derived from either the unsupervised consensus clustering or stratification by *IDH* mutation status and *PER* expression level. Comparisons of clusters Circadian1 versus Circadian2, and Circadian3 versus Circadian4, revealed that Circadian2 and Circadian4 (characterized by lower *PER* expression compared to Circadian1 and Circadian3, respectively) overexpressed immune signaling pathway genes (Figure 3A). Examples of these pathways include granulocyte adhesion and diapedesis, dendritic cell maturation, crosstalk between dendritic cells and natural killer cells, and IL-10 signaling, among others. Functional enrichment analysis performed on the samples first stratified by the *IDH* mutational status then the *PER* expression levels similarly resulted in low *PER* expression being associated with an enrichment of immune signaling regardless of the *IDH* mutational status (Figure 3B). Within the stratified analysis, we further assessed the functional enrichment by 1p/19q co-deletion status. We found the enrichment of immune signaling in 1p/19q intact tumors with low *PER* expression (Figure 3C, right panel) but not in the 1p/19q co-deleted tumors (Figure 3C, left panel).

The discovery that samples with low *PER* expression were characterized by upregulation of immune pathways led us to consider whether these differences could be an artifact of the “bulk” nature of the profiled samples, which included a varied mixture of microenvironment components and cells. To investigate this hypothesis, we downloaded computed xCell scores—estimates of the contribution of different cell types in bulk tumors derived through a transcriptomic deconvolution algorithm [49]—and compared the immune scores and stromal scores across the clusters Circadian1–Circadian4 (Figure S4). Differences in these scores could be observed when comparing clusters with different *IDH* mutation status composition; however, comparisons of clusters with similar composition (Circadian1 versus Circadian2 and Circadian3 versus Circadian4) showed no differences in stromal scores, and only the pair of Circadian3 versus Circadian4 (enriched for *IDH* wild-type samples) was significantly different in the immune score (Table S4). Since Circadian1 and Circadian2 were not significantly different, we concluded that the cellular composition of the samples could not be fully responsible for the upregulation of immune signaling that we observed in low *PER* clusters.

We also investigated whether the differences in *PER* expression could be due to changes in DNA methylation, hypothesizing that a lower *PER* expression might be a result of higher levels of DNA methylation in these genes. We compared the gene level methylation of the *PER* genes across the identified clusters Circadian1–Circadian4. However, we did not find gene-level methylation differences that could explain the lower *PER* expression through the epigenetic silencing of these genes (Figure S5).

### 2.4. Clock Genes Are Differentially Expressed across Anatomical Tumor Locations

**Figure 4:**
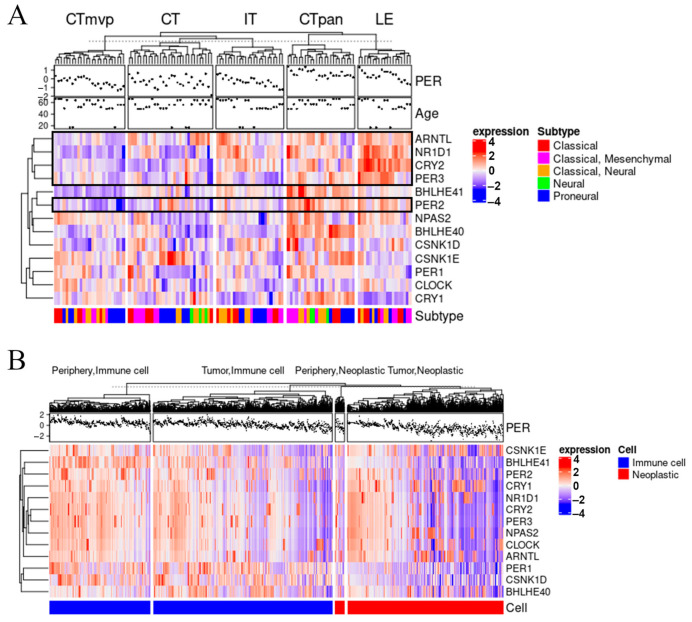
Differential clock gene expression by tumor location and cell type. (**A**) Heatmap generated from the IvyGap dataset showing the upregulation (red) and downregulation (blue) of clock genes across anatomical locations of glioblastoma tumors: Leading Edge (LE), Pseudopalisading cells around necrosis (CTpan), Infiltrating Tumor (IT), Cellular Tumor (CT), and Microvascular proliferation (CTmvp). Core clock genes are highlighted in the black boxes. (**B**) Heatmap generated from the Darmanis dataset showing the upregulation (red) and downregulation (blue) of clock genes in neoplastic and immune cells in the core and the periphery of the tumor.

The enrichment of immune signaling pathways in samples with low *PER* expression (Figure 3) indicates that *PER* expression might affect or be affected by the immune microenvironment in glioma. The tumor microenvironment (TME) in glioma is heterogeneous [50,51,52] and characterized by a number of environmental pressures that can directly impact gene expression [53]. To assess the potential relationship between the tumor microenvironment and *PER* gene expression, we analyzed the clock gene expression in multiple regions within the tumor using the IvyGap dataset [37]. IvyGap contains transcriptomic profiles of samples taken from multiple distinct anatomic regions within the same patient tumor. Specifically, the dataset includes gene expression data representing 41 patients with samples taken from the following anatomic regions (with well-characterized histopathologic features) present in *IDH* wild-type glioblastoma (GBM): cellular tumor (CT), leading edge (LE), infiltrating tumor (IT), pseudopalisading region around necrosis (CTpan), and microvascular proliferation (CTmvp). We found that the intratumor location affects the core clock gene expression (Figure 4A). The core clock gene expression, including the *PER*, were found to be higher in the CTpan and LE regions and lower in the CT and CTmvp regions (Figure 4A). These results suggest that the tumor microenvironment impacts the clock gene expression.

### 2.5. Clock Gene Expression Varies across Cellular Subpopulations within Glioma Tumors

The higher immune score as defined by overexpression of immune signaling pathways in low *PER* TCGA clusters (Circadian2 and Circadian4) as compared to the high *PER* counterparts (Circadian1 and Circadian3) and the differential expression of *PER* based on tumor location raised the question of whether different cellular types within the glioma tumors differ in their expression of clock genes. We investigated this hypothesis using data from Darmanis and colleagues [38], who performed single-cell RNA-sequencing to assess the glioma microenvironment landscape including tumor and stromal cells from the core and periphery of (4 *IDH* wild-type) glioblastoma patient tumors. Supervised clustering of the profiles of neoplastic and immune cell populations revealed a pattern of upregulation of core clock genes in immune cells compared to neoplastic cells; these genes included *PER1–3*. Interestingly, a gradient of expression for these genes was observed within the immune cells, with cells from the periphery displaying a stronger upregulation of the core clock genes compared to the cells from the core of the tumor (Figure 4B). This was harder to assess for neoplastic cells given the relatively small number of neoplastic cells in the tumor periphery that were profiled. Together with the IvyGap dataset, these findings suggest both the location of the cells in relation to the tumor and the classification of the cell type are important factors that should be further studied.

**Figure 5:**
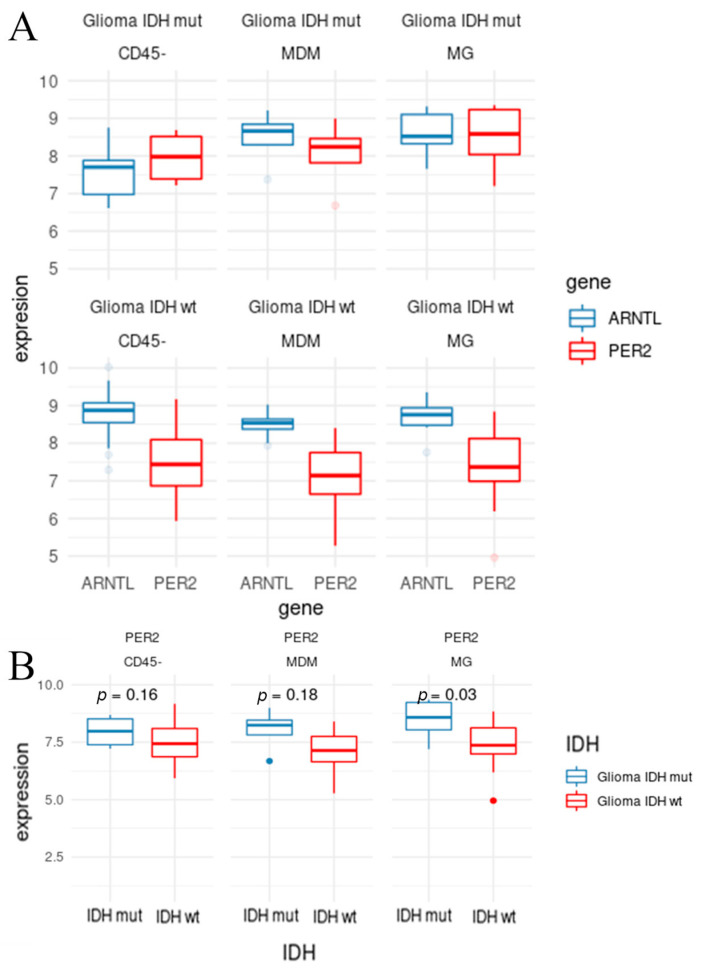
Clock gene expression in the immune cell composition of gliomas by IDH status. (**A**) Boxplots show expression levels of *PER2* (red) and *ARNTL* (blue) for microglia (MG), myeloid-derived macrophages (MDM), and CD45 negative (CD45-) cells in *IDH*-mutated (Glioma *IDH* mut) and wild-type tumors (Glioma *IDH* wt). (**B**) Boxplot showing the expression levels of *PER2* for *IDH*-mutated (blue) and *IDH* wild-type (red) tumors in MG, MDM, and CD45 cells. The *t*-test comparisons show that the *PER2* expression levels in MG are significantly lower in *IDH* wild-type compared to MG in *IDH*-mutated tumors.

We expanded our investigation of cell-specific effects on the expression of core clock genes in tumors (Figure S5) by taking into account the *IDH* status using the data published by Klemm and colleagues [39]. This published dataset represents tumor samples from 24 patients, seven of which had *IDH*-mutated tumors, and the remainder had *IDH* wild-type tumors. We examined the impact of *PER2* expression in these cells, as well as *ARNTL*, as it is often antiphasic to the period expression in vitro (Figure 5A). We assessed the *PER2* expression levels in microglia (MG), myeloid-derived macrophages (MDM), and CD45-negative cells (CD45n) between the *IDH*-mutant and *IDH* wild-type tumors (Figure 5). The *PER2* expression is lower across all cell types in the *IDH* wild-type tumors; however, the difference is only statistically significant in the MG (*p* = 0.03).

## 3. Discussion

The discovery of targetable universal mechanisms that affect cancer growth remains an elusive challenge but would provide a promising vehicle for identifying treatments of aggressive cancers, such as glioma. Alterations in circadian clock genes have been directly linked to clinical outcomes such as treatment toxicities [31] and overall survival [30,34] in glioma patients. Even though *IDH* mutational status has distinct biologic and prognostic importance in malignant gliomas, to our knowledge, no prior clock gene/glioma studies have taken into consideration the *IDH* mutational status. Analyzing molecular profiles from patient tumors available in several public datasets, we discovered distinct transcriptional subtypes based on 13 circadian clock genes and that these subtypes could not be explained by the *IDH* mutation status alone but may be reflective of different disease states. This hypothesis was supported by the finding that the prognosis of the patients whose tumors displayed different clock subtypes were also found to be different. Low expression of the period genes (*PER*) was associated with poor prognosis in both *IDH*-mutant and wild-type groups of astrocytic lineage, although the association did not rise to statistical significance for the oligodendroglial (1p/19q co-deleted) lineage. Importantly, absolute *PER* expression levels were not found to be directly related to prognosis: for example, in the TCGA clustering analysis, patients from cluster Circadian3 enriched for *IDH* wild-type samples had a higher expression of *PER* but worse prognosis than patients from cluster Circadian1 enriched for *IDH*-mutated gliomas (Figure S2). While this result is expected due to the known advantage of *IDH* mutations on survival and the lower age of the patients harboring *IDH*-mutant tumors, it also highlights the need to assess *PER* expression relative to the *IDH* mutant class of the tumors.

Our functional pathway analysis of cohorts with lower *PER* expression and worse relative survival showed the upregulation of immune signaling pathways and suggested that immunological mechanisms may be responsible for these differences in survival. In this context, circadian rhythms directly modulate the immune system in the periphery, regulating a myriad of immune functions, such as cytokine and chemokine secretion [54,55]. Disruption of these rhythms caused by chronic jetlag [56,57] or the genetic deletion of core clock genes [58,59] has a negative impact on the functionality of immune cells and can lead to premature death. Age is another factor that can disrupt clock gene expression and circadian rhythms at the organism level leading to premature death [60]. Although our analysis indicates that the *PER* expression level is an additional independent prognostic factor, alongside age and *IDH* mutation status, the untangling of any interaction between these factors deserves further examination through time series studies. An intriguing connection between age and period length at the cellular level has been recently proposed by Li et al., who examined the circadian period length in single cells and clonal cell lines and found that a longer period is associated with the increased intercellular heterogeneity and transcriptomic noise often observed in aging cells and cancer [61]. Within solid cancers [62,63,64], including glioma [30], circadian misalignment is associated with increased tumor growth rate and decreased survival. These findings have been hypothesized to be caused by suppression of the circadian-related immune function. A recent publication tested the hypothesis in a melanoma mouse model [65], demonstrating a loss of daily rhythms in macrophage infiltration within the tumor microenvironment and spleen under chronic jetlag conditions. By analyzing the IvyGap dataset, we found that there is higher expression of core clock genes, including *PER*, in specific anatomical tumor microenvironments: the leading edge and the pseudopalisading region around the necrosis of the tumor. These subregions are associated with different immune signatures and oxygenation levels [66]. Greater interrogation of the specific immune cells within these regions would provide insight into the negative circadian effects caused by the hypoxia conditions [67] or tumor cell proximity.

Most cells within the body express circadian rhythms in the transcriptome; however, the genes that are regulated and their pattern of expression are dependent on the tissue type [28]. Within the brain, the regions can express different and sometimes antiphasic patterns of clock and clock-controlled genes compared to the master biological clock found in the suprachiasmatic nucleus [28,68,69]. In vitro, the different cell types that compose the brain, including neurons [70], glial [71], epithelial [72], and immune cells [73], have been shown to express rhythms of clock genes. When examining circadian rhythms in the glioma, tumor cells have been the primary and only focus of cell culture works. These studies have demonstrated the importance of clock genes on tumor growth [30] and the optimal timing of treatment, including temozolomide [74] and radiation [75,76]. Additionally, our findings here demonstrate that clock genes of immune cells found in the tumor microenvironment are different based on *IDH* mutational status, specifically microglia. Our data do not include a time series, so alterations in circadian rhythms cannot be deduced. The bulk tissue isolated from patients often does not have time stamp information on the collection times, and this is a major limitation of genomic studies using publicly available datasets. However, the rewiring of circadian rhythms by other solid tumors has been shown within support cells found in the local microenvironment [77] and systemically, impacting circadian metabolic function in the liver [78]. Glioma cells have been shown to directly suppress the immune function in the periphery, which demonstrates the capability of tumors to impact distant foci and suggests a mode by which circadian rhythms may be altered within immune cells [79]. Recently, the relationship between circadian disruption in tumors and microglia invasion into the glioma was explored in mice and cell cultures by Chen et al. [80]. The authors demonstrated that the suppression of *CLOCK* and *BMAL* in tumor cells improved the survival and reduced migration of the microglia. These findings, however, only focused on the tumor rhythms and did not examine the impact of clock genes in the microglia. Understanding the circadian rhythms within and beyond the neoplastic cells will be the key to developing clock gene-based strategies to reduce the treatment side effects and improve survivorship. Future works should broaden the focus of clock genes in glioma research to include the microenvironment and its relationship to tumor cells.

## 4. Materials and Methods

### 4.1. Data Sources

RNA-seq expression data from TCGA (668 grades 2–4) primary glioma samples were downloaded using the R TCGAbiolinks library [81,82]. The data were preprocessed, normalized, and filtered as recommended in R’s TCGAWorkflow library [83].

Array hybridization mRNA expression data and progression-free survival (PFS) patient information from 195 patients diagnosed with low-grade (2) glioma enrolled in the EORTC22033-26033 clinical trial were downloaded from Gene Expression Omnibus (GEO) (accession: GSE107850 [36]).

RNA-seq profiles for 270 laser-microdissected samples from 41 GBM tumors were downloaded from the Allen Institute’s Ivy Glioblastoma Atlas Project (Ivy-GAP) (http://glioblastoma.alleninstitute.org/, accessed on 24 January 2018, GSE107559) [37]. Samples were collected from different anatomical locations of the tumor, including cellular tumor (CT, 30 samples), leading edge (LE, 19), infiltrating tumor (IT, 24), microvascular proliferation (CTmvp, 25), and pseudopalisading region around necrosis (CTpan, 24). The data were preprocessed, normalized, and filtered using R’s TCGAWorkflow library.

scRNA-seq data of 4028 single cells from 4 primary GBM tumors were downloaded from GEO (accession: GSE84465; [38]). Samples from each patient were taken from the tumor core and peritumoral tissue (cortex). The data were normalized using quantile normalization. Individual gene queries were performed using the online data portal (http://gbmseq.org/, accessed on 16 January 2021).

RNA-seq expression data of immune cell populations within the microenvironment of primary glioma samples were downloaded from Klemm et al. [39]. Cell sorting of clinical samples was performed prior to sequencing for identification of immune cell populations. The dataset represented 48 patients diagnosed with brain tumors, 24 of which were diagnosed with glioma grades 2–4. The glioma patient cohort included 7 patients with *IDH* mutated tumors and 17 with *IDH* wild-type tumors. (https://joycelab.shinyapps.io/braintime/, accessed on 30 April 2020).

xCell bulk tumor deconvolution scores for the TCGA samples were downloaded from the xCell portal (https://xcell.ucsf.edu/, accessed on 5 August 2020) [49]. The predicted, sample-level Immune Score and Stroma Score were used to assess the overall differences in the contribution of immune and stroma elements to the bulk samples from the discovered cohorts of interest.

DNA methylation scores for TCGA samples were downloaded from Wanderer (http://maplab.imppc.org/wanderer/, accessed on 12 November 2020). Gene-level scores were computed by averaging the corresponding CpG island-level scores [84].

### 4.2. Hierarchical Clustering

Data were analyzed using R’s TCGAbiolinks and ConsensusClusterPlus libraries. Unsupervised consensus clustering of the samples was performed based on the transcriptomic profiles of 13 clock genes (*BHLHE41*, *BHLHE40*, *CLOCK*, *NPAS2*, *ARNT*L, *NR1D1*, *CRY2*, *PER3*, *PER2*, *PER1*, *CSNK1E*, *CRY1*, and *CSNK1D*); the genes used are members of the KEGG_CIRCADIAN_RHYTHM_MAMMAL pathway of the Kyoto Encyclopedia of Genes and Genomes (KEGG) knowledgebase and were obtained from the Molecular Signatures database (MSigDB) (http://www.gsea-msigdb.org/gsea/msigdb/index.jsp, accessed on 25 January 2018) [42,85,86]. Clustering into 2–7 groups was considered. Clustering into 4 groups was found to be in the optimal range as assessed using the ConsensusClusterPlus [87] analysis of the stability of cluster membership over multiple runs of the algorithm, as well as the drop in improvement of the Cumulative Distribution Function with each addition to the number of clusters. A similar analysis was performed on the LGG dataset from Gao et al. [36].

Unless otherwise specified, R’s ComplexHeatmap [88] library was used for visualization of all hierarchical clusters, including all heatmaps depicting transcriptomic differences across cohorts, tumor locations, cell types, and xCell scores.

### 4.3. Survival Analysis

Kaplan–Meier analysis was performed to assess any differences in overall survival (OS) between sample clusters discovered through unsupervised clustering of the TCGA samples. For the LGG cohorts, in the absence of OS information, PFS differences were instead assessed. The significance of the differences was determined using log-rank tests; *p* < 0.05 were considered significant in pairwise comparisons. Analogous analyses were performed for the cohorts stratified by *IDH* mutation status, 1p19q codeletion status, and *PER1/2/3* expression levels dichotomized around the median expression.

Cox-proportional hazards analysis was performed to assess the predictive power of clock gene expression levels on OS of TCGA patients and PFS for the LGG cohort. Models were constructed with covariates age at diagnosis, *IDH* mutational status (and for TCGA 1p/19q codeletion status), and combined expression of *PER*(1/2/3) (averaged per each sample), as well as with covariates age at diagnosis, *IDH* mutational status (and for TCGA 1p/19q codeletion status), and all 13 clock genes as separate covariates. Likelihood ratio test of the overall model was assessed, as well as the Hazard Ratio (HR) of individual covariates. HR with a 95% Confidence Interval not crossing 1 were considered significant (or interchangeably, the individual covariates’ contributions to the model were considered significant when associated *p* < 0.05).

### 4.4. Differential Gene Expression and Functional Pathway Enrichment Analysis

Differential gene expression analysis between TCGA clusters was performed using the TCGAbiolinks library, whose functionality is tailored to the differential gene expression analysis of samples with experimental and batching characteristics of TCGA. Two sets of differential expression analyses were performed: between clusters discovered through consensus clustering (here, the comparisons were done for clusters with similar molecular backgrounds, comparing the clusters enriched for *IDH*-mutant samples to each other, and comparing the clusters enriched for *IDH* wild-type samples to each other), as well as between clusters that have the same *IDH* mutational status but are split according to the median expression of *PER*. Fold change (FC) > 1.5 and adjusted *p* < 0.05 were used as the criteria for a significant differential expression of the genes.

Gene lists obtained from the differential gene expression analysis were submitted for functional pathway enrichment analysis using TCGAbiolinks and annotations from the Database for Annotation, Visualization and Integrated Discovery (DAVID) knowledgebase [89,90]. Pathways with adjusted *p* < 0.05 of enrichment were considered significantly enriched.

### 4.5. Other Comparisons

Comparisons of the Immune and Stroma Scores between the TCGA clusters were assessed using R’s limma package [91]: fitting a linear model, computing moderated *t*-statistics, moderated F-statistic, and log-odds of differential expression. The results were adjusted for multiple comparisons, and an adjusted *p* < 0.05 was used as the criterion of significance.

Individual gene expression differences were depicted using R’s ggplot2 boxplot functionality and assessed using *t*-tests through the ggpubr library. Barplots of gene expression were generated by running queries at http://gbmseq.org/ (accessed on 16 January 2021) for the Darmanis et al. dataset [38] and at https://joycelab.shinyapps.io/braintime/ (accessed on 30 April 2020) for the Klemm et al. dataset [39]. Where *t*-tests were used, *p* < 0.05 was considered to indicate statically significant differences.

## 5. Conclusions

Our results highlighted the association of the circadian clock gene expression and glioma patient outcomes across the glioma landscape and independent of the *IDH* mutational status of glial tumors. Importantly, we showed a complex interplay of the tumor microenvironment and the circadian clock, demonstrating that expression of the clock genes is highly plastic and varied across tumor regions and cell types. Taken together, our findings support that circadian clock transcriptomics are directly reflective of clinically relevant disease states and therefore represent important potential biomarkers and targets for therapeutic interventions.

## Figures and Tables

**Table 1:** Unsupervised consensus clustering based on 13 clock genes.

Demographic and Molecular Characteristics	Circadian1	Circadian2	Circadian3	Circadian4
*IDH* mutation status				
Mutant	94.95	61.73	47.37	18.95
WT	4.69	37.65	52.63	77.78
NA	0.36	0.62	0	3.27
*IDH* codel subtype				
*IDH*mut-codel	43.68	22.84	9.21	2.61
*IDH*mut-non-codel	51.26	38.89	36.84	16.34
*IDH*wt	4.69	37.04	52.63	75.82
NA	0.36	1.23	1.32	5.23
Pan glioma methylation subtype				
LGm1	7.27	15.03	6.58	2.96
LGm2	53.82	35.29	35.53	19.26
LGm3	35.27	13.73	5.26	0.74
LGm4	1.45	7.19	25.00	23.70
LGm5	1.09	16.99	22.37	42.96
LGm6	1.09	11.76	5.26	10.37
Original TCGA subtype				
Classical	0.36	6.21	7.89	14.09
G-CIMP	1.09	1.24	3.95	0
*IDH*mut-codel	43.84	22.98	9.21	2.68
*IDH*mut-non-codel	50.36	37.27	32.89	16.78
*IDH*wt	2.17	14.91	34.21	26.17
Mesenchymal	0	3.11	5.26	26.85
Neural	0	6.21	3.95	8.72
Proneural	2.17	8.07	2.63	4.7
Gender				
Female	40.82	47.37	43.75	37.41
Male	59.18	52.63	56.25	62.59
Average age	42.29	47.43	47.77	55.43

## Data Availability

All datasets used in this study are publicly available.

## Supplementary Materials

The following are available online at https://www.mdpi.com/article/10.3390/cancers13112756/s1: Figure S1: Graphs demonstrating the coordinated patterns of expression of clock genes in the TCGA data and unsupervised consensus clusters. Figure S2: Clock gene expression in the unsupervised clustering analysis. Figure S3: Prognostic relevance of *PER* in unsupervised clusters for LGG dataset. Figure S4: Contribution of different cell types in the bulk tumor derived through a transcriptomic deconvolution algorithm. Figure S5: Differences in *PER2* and *ARNTL* expression patterns between different cells of the tumor microenvironment. Figure S6: Clock gene expression in the subgroups separated by methylation status. Table S1. Differences in clock gene expression between high and low *PER*-expressing circadian clusters with predominantly *IDH* mutant or wild type. Table S2: Kaplan–Meier analysis of the overall survival between unsupervised consensus clusters of TCGA data. Table S3: Summary of the full Cox model of the 13 core clock gene expression and reduced model of significant clock genes for the TCGA dataset. Table S4: Tumor composition analysis by deconvolution.

## References

[1] Louis D.N., Ohgaki H., Wiestler O.D., Cavenee W.K., Burger P.C., Jouvet A., Scheithauer B.W., Kleihues P. (2007). The 2007 WHO Classification of Tumours of the Central Nervous System. Acta Neuropathol..

[2] Louis D.N., Perry A., Reifenberger G., Von Deimling A., Figarella-Branger D., Cavenee W.K., Ohgaki H., Wiestler O.D., Kleihues P., Ellison D.W. (2016). The 2016 World Health Organization Classification of Tumors of the Central Nervous System: A summary. Acta Neuropathol..

[3] Ostrom Q.T., Cioffi G., Gittleman H., Patil N., Waite K., Kruchko C., Barnholtz-Sloan J.S. (2019). CBTRUS Statistical Report: Primary Brain and Other Central Nervous System Tumors Diagnosed in the United States in 2012–2016. Neuro Oncol..

[4] Morgan L.L. (2015). The epidemiology of glioma in adults: A “state of the science” review. Neuro Oncol..

[5] Hanif F., Muzaffar K., Perveen K., Malhi S.M., Simjee S.U. (2017). Glioblastoma Multiforme: A Review of its Epidemiology and Pathogenesis through Clinical Presentation and Treatment. Asian Pac. J. Cancer Prev..

[6] Lamborn K.R., Chang S.M., Prados M.D. (2004). Prognostic factors for survival of patients with glioblastoma: Recursive partitioning analysis. Neuro Oncol..

[7] Ceccarelli M., Barthel F., Malta T.M., Sabedot T.S., Salama S., Murray B.A., Morozova O., Newton Y., Radenbaugh A., Pagnotta S.M. (2016). Molecular Profiling Reveals Biologically Discrete Subsets and Pathways of Progression in Diffuse Glioma. Cell.

[8] Gravendeel L.A.M., Kouwenhoven M.C.M., Gevaert O., De Rooi J.J., Stubbs A.P., Duijm J.E., Daemen A., Bleeker F.E., Bralten L.B.C., Kloosterhof N.K. (2009). Intrinsic Gene Expression Profiles of Gliomas Are a Better Predictor of Survival than Histology. Cancer Res..

[9] Radke J., Koch A., Pritsch F., Schumann E., Misch M., Hempt C., Lenz K., Löbel F., Paschereit F., Heppner F.L. (2019). Predictive MGMT status in a homogeneous cohort of IDH wildtype glioblastoma patients. Acta Neuropathol. Commun..

[10] LaBreche K., Kinnersley B., Berzero G., Di Stefano A.L., Rahimian A., Detrait I., Marie Y., Grenier-Boley B., Hoang-Xuan K., Delattre J.-Y. (2018). Diffuse gliomas classified by 1p/19q co-deletion, TERT promoter and IDH mutation status are associated with specific genetic risk loci. Acta Neuropathol..

[11] Madala H.R., Punganuru S.R., Arutla V., Misra S., Thomas T.J., Srivenugopal K.S. (2018). Beyond Brooding on Oncometabolic Havoc in IDH-Mutant Gliomas and AML: Current and Future Therapeutic Strategies. Cancers.

[12] D’Alessio A., Proietti G., Sica G., Scicchitano B.M. (2019). Pathological and Molecular Features of Glioblastoma and Its Peritumoral Tissue. Cancers.

[13] Huang J., Yu J., Tu L., Huang N., Li H., Luo Y. (2019). Isocitrate Dehydrogenase Mutations in Glioma: From Basic Discovery to Therapeutics Development. Front. Oncol..

[14] Romanidou O., Kotoula V., Fountzilas G. (2018). Bridging Cancer Biology with the Clinic: Comprehending and Exploiting IDH Gene Mutations in Gliomas. Cancer Genom. Proteom..

[15] Wang P., Wu J., Ma S., Zhang L., Yao J., Hoadley K.A., Wilkerson M.D., Perou C., Guan K.-L., Ye D. (2015). Oncometabolite D-2-Hydroxyglutarate Inhibits ALKBH DNA Repair Enzymes and Sensitizes IDH Mutant Cells to Alkylating Agents. Cell Rep..

[16] Sulkowski P.L., Corso C.D., Robinson N.D., Scanlon S.E., Purshouse K.R., Bai H., Liu Y., Sundaram R.K., Hegan D.C., Fons N. (2017). 2-Hydroxyglutarate produced by neomorphic IDH mutations suppresses homologous recombination and induces PARP inhibitor sensitivity. Sci. Transl. Med..

[17] Chaumeil M.M., Radoul M., Najac C., Eriksson P., Viswanath P., Blough M.D., Chesnelong C., Luchman H.A., Cairncross J.G., Ronen S.M. (2016). Hyperpolarized 13 C MR imaging detects no lactate production in mutant IDH1 gliomas: Implications for diagnosis and response monitoring. NeuroImage Clin..

[18] Ruiz-Rodado V., Malta T.M., Seki T., Lita A., Dowdy T., Celiku O., Cavazos-Saldana A., Li A., Liu Y., Han S. (2020). Metabolic reprogramming associated with aggressiveness occurs in the G-CIMP-high molecular subtypes of IDH1mut lower grade gliomas. Neuro Oncol..

[19] Davidson A.J., Castañón-Cervantes O., Stephan F.K. (2004). Daily oscillations in liver function: Diurnal vs circadian rhythmicity. Liver Int..

[20] Reinke H., Asher G. (2019). Crosstalk between metabolism and circadian clocks. Nat. Rev. Mol. Cell Biol..

[21] Boucher H., Vanneaux V., Domet T., Parouchev A., Larghero J. (2016). Circadian Clock Genes Modulate Human Bone Marrow Mesenchymal Stem Cell Differentiation, Migration and Cell Cycle. PLoS ONE.

[22] Sancar A., Lindsey-Boltz L., Kang T.-H., Reardon J.T., Lee J.H., Ozturk N. (2010). Circadian clock control of the cellular response to DNA damage. FEBS Lett..

[23] Partch C.L., Green C.B., Takahashi J.S. (2014). Molecular architecture of the mammalian circadian clock. Trends Cell Biol..

[24] Mohawk J.A., Green C.B., Takahashi J.S. (2012). Central and peripheral circadian clocks in mammals. Annu. Rev. Neurosci..

[25] Xie Y., Tang Q., Chen G., Xie M., Yu S., Zhao J., Chen L. (2019). New Insights into the Circadian Rhythm and Its Related Diseases. Front. Physiol..

[26] Ono D., Honma S., Nakajima Y., Kuroda S., Enoki R., Honma K.-I. (2017). Dissociation of Per1 and Bmal1 circadian rhythms in the suprachiasmatic nucleus in parallel with behavioral outputs. Proc. Natl. Acad. Sci. USA.

[27] Costa S.S.F., Robinson-Rechavi M., A Ripperger J. (2020). Single-cell transcriptomics allows novel insights into aging and circadian processes. Brief. Funct. Genom..

[28] Mure L.S., Le H.D., Benegiamo G., Chang M.W., Rios L., Jillani N., Ngotho M., Kariuki T., Dkhissi-Benyahya O., Cooper H.M. (2018). Diurnal transcriptome atlas of a primate across major neural and peripheral tissues. Science.

[29] Sulli G., Lam M.T.Y., Panda S. (2019). Interplay between Circadian Clock and Cancer: New Frontiers for Cancer Treatment. Trends Cancer.

[30] Dong Z., Zhang G., Qu M., Gimple R.C., Wu Q., Qiu Z., Prager B.C., Wang X., Kim L.J., Morton A.R. (2019). Targeting Glioblastoma Stem Cells through Disruption of the Circadian Clock. Cancer Discov..

[31] Armstrong T.S., Vera E., Zhou R., Acquaye A.A., Sullaway C.M., Berger A.M., Breton G., Mahajan A., Wefel J.S., Gilbert M.R. (2017). Association of genetic variants with fatigue in patients with malignant glioma. Neuro Oncol. Pract..

[32] Sun X.-X., Yu Q. (2015). Intra-tumor heterogeneity of cancer cells and its implications for cancer treatment. Acta Pharmacol. Sin..

[33] Patel A.P., Tirosh I., Trombetta J.J., Shalek A.K., Gillespie S.M., Wakimoto H., Cahill D.P., Nahed B., Curry W.T., Martuza R.L. (2014). Single-cell RNA-seq highlights intratumoral heterogeneity in primary glioblastoma. Science.

[34] Ye Y., Xiang Y., Ozguc F.M., Kim Y., Liu C.-J., Park P.K., Hu Q., Diao L., Lou Y., Lin C. (2018). The Genomic Landscape and Pharmacogenomic Interactions of Clock Genes in Cancer Chronotherapy. Cell Syst..

[35] Weinstein J.N., Collisson E.A., Mills G.B., Shaw K.R., Ozenberger B.A., Ellrott K., Shmulevich I., Sander C., Stuart J.M., The Cancer Genome Atlas Research Network (2013). The Cancer Genome Atlas Pan-Cancer analysis project. Nat. Genet..

[36] Gao Y., Weenink B., Bent M.V.D., Erdem-Eraslan L., Kros J., Smitt P.S., Hoang-Xuan K., Brandes A., Vos M., Dhermain F. (2018). Expression-based intrinsic glioma subtypes are prognostic in low-grade gliomas of the EORTC22033-26033 clinical trial. Eur. J. Cancer.

[37] Puchalski R.B., Shah N., Miller J., Dalley R., Nomura S.R., Yoon J.-G., Smith K.A., Lankerovich M., Bertagnolli D., Bickley K. (2018). An anatomic transcriptional atlas of human glioblastoma. Science.

[38] Darmanis S., Sloan S.A., Croote D., Mignardi M., Chernikova S., Samghababi P., Zhang Y., Neff N., Kowarsky M., Caneda C. (2017). Single-Cell RNA-Seq Analysis of Infiltrating Neoplastic Cells at the Migrating Front of Human Glioblastoma. Cell Rep..

[39] Klemm F., Maas R.R., Bowman R.L., Kornete M., Soukup K., Nassiri S., Brouland J.-P., Iacobuzio-Donahue C.A., Brennan C., Tabar V. (2020). Interrogation of the Microenvironmental Landscape in Brain Tumors Reveals Disease-Specific Alterations of Immune Cells. Cell.

[40] Cancer Genome Atlas Research Network (2008). Comprehensive genomic characterization defines human glioblastoma genes and core pathways. Nature.

[41] The Cancer Genome Atlas Research Network (2015). Comprehensive, Integrative Genomic Analysis of Diffuse Lower-Grade Gliomas. N. Engl. J. Med..

[42] Ogata H., Goto S., Sato K., Fujibuchi W., Bono H., Kanehisa M. (1999). KEGG: Kyoto Encyclopedia of Genes and Genomes. Nucleic Acids Res..

[43] Capper D., Jones D.T.W., Sill M., Hovestadt V., Schrimpf D., Sturm D., Koelsche C., Sahm F., Chavez L., Reuss D.E. (2018). DNA methylation-based classification of central nervous system tumours. Nature.

[44] Capper D., Stichel D., Sahm F., Jones D.T.W., Schrimpf D., Sill M., Schmid S., Hovestadt V., Reuss D.E., Koelsche C. (2018). Practical implementation of DNA methylation and copy-number-based CNS tumor diagnostics: The Heidelberg experience. Acta Neuropathol..

[45] Xiong H., Yang Y., Yang K., Zhao D., Tang H., Ran X. (2017). Loss of the clock gene PER2 is associated with cancer development and altered expression of important tumor-related genes in oral cancer. Int. J. Oncol..

[46] Fu L., Pelicano H., Liu J., Huang P., Lee C.C. (2002). The circadian gene period2 plays an important role in tumor suppression and DNA damage response in vivo. Cell.

[47] Lin Y., Chang J.H., Yeh K., Yang M., Liu T., Lin S., Su W., Chang J. (2008). Disturbance of circadian gene expression in hepatocellular carcinoma. Mol. Carcinog..

[48] Wang Q., Ao Y., Yang K., Tang H., Chen D. (2016). Circadian clock gene Per2 plays an important role in cell proliferation, apoptosis and cell cycle progression in human oral squamous cell carcinoma. Oncol. Rep..

[49] Aran D., Hu Z., Butte A.J. (2017). xCell: Digitally portraying the tissue cellular heterogeneity landscape. Genome Biol..

[50] De Vleeschouwer S., Bergers G., De Vleeschouwer S. (2017). Glioblastoma: To Target the Tumor Cell or the Microenvironment?. Glioblastoma.

[51] Schiffer D., Annovazzi L., Casalone C., Corona C., Mellai M. (2018). Glioblastoma: Microenvironment and Niche Concept. Cancers.

[52] Antunes A.R.P., Scheyltjens I., Duerinck J., Neyns B., Movahedi K., A Van Ginderachter J. (2020). Understanding the glioblastoma immune microenvironment as basis for the development of new immunotherapeutic strategies. eLife.

[53] Cooper L.A., Gutman D.A., Chisolm C., Appin C., Kong J., Rong Y., Kurc T., Van Meir E.G., Saltz J.H., Moreno C. (2012). The Tumor Microenvironment Strongly Impacts Master Transcriptional Regulators and Gene Expression Class of Glioblastoma. Am. J. Pathol..

[54] Yeung J., Mermet J., Jouffe C., Marquis J., Charpagne A., Gachon F., Naef F. (2018). Transcription factor activity rhythms and tissue-specific chromatin interactions explain circadian gene expression across organs. Genome Res..

[55] Arjona A., Silver A.C., Walker W.E., Fikrig E. (2012). Immunity’s fourth dimension: Approaching the circadian-immune connection. Trends Immunol..

[56] Castanon-Cervantes O., Wu M., Ehlen J.C., Paul K., Gamble K.L., Johnson R.L., Besing R.C., Menaker M., Gewirtz A.T., Davidson A.J. (2010). Dysregulation of Inflammatory Responses by Chronic Circadian Disruption. J. Immunol..

[57] Inokawa H., Umemura Y., Shimba A., Kawakami E., Koike N., Tsuchiya Y., Ohashi M., Minami Y., Cui G., Asahi T. (2020). Chronic circadian misalignment accelerates immune senescence and abbreviates lifespan in mice. Sci. Rep..

[58] Kitchen G.B., Cunningham P., Poolman T.M., Iqbal M., Maidstone R., Baxter M., Bagnall J., Begley N., Saer B., Hussell T. (2020). The clock gene Bmal1 inhibits macrophage motility, phagocytosis, and impairs defense against pneumonia. Proc. Natl. Acad. Sci. USA.

[59] Hergenhan S., Holtkamp S., Scheiermann C. (2020). Molecular Interactions Between Components of the Circadian Clock and the Immune System. J. Mol. Biol..

[60] Shuboni-Mulligan D.D., Young D.L., Minyety J.D.L.C., Vera E., Munasinghe J., Gall A.J., Gilbert M.R., Armstrong T.S., Smart D.K. (2021). Impact of age on the circadian visual system and the sleep-wake cycle in mus musculus. NPJ Aging Mech. Dis..

[61] Li Y., Shan Y., Desai R.V., Cox K.H., Weinberger L.S., Takahashi J.S. (2020). Noise-driven cellular heterogeneity in circadian periodicity. Proc. Natl. Acad. Sci. USA.

[62] Filipski E., Delaunay F., King V.M., Wu M.-W., Claustrat B., Gréchez-Cassiau A., Guettier C., Hastings M.H., Francis L. (2004). Effects of Chronic Jet Lag on Tumor Progression in Mice. Cancer Res..

[63] Papagiannakopoulos T., Bauer M.R., Davidson S.M., Heimann M., Subbaraj L., Bhutkar A., Bartlebaugh J., Heiden M.G.V., Jacks T. (2016). Circadian Rhythm Disruption Promotes Lung Tumorigenesis. Cell Metab..

[64] Hadadi E., Taylor W., Li X.-M., Aslan Y., Villote M., Rivière J., Duvallet G., Auriau C., Dulong S., Raymond-Letron I. (2020). Chronic circadian disruption modulates breast cancer stemness and immune microenvironment to drive metastasis in mice. Nat. Commun..

[65] Aiello I., Fedele M.L.M., Román F., Marpegan L., Caldart C., Chiesa J.J., Golombek D.A., Finkielstein C.V., Paladino N. (2020). Circadian disruption promotes tumor-immune microenvironment remodeling favoring tumor cell proliferation. Sci. Adv..

[66] Celiku O., Gilbert M.R., Lavi O. (2019). Computational modeling demonstrates that glioblastoma cells can survive spatial environmental challenges through exploratory adaptation. Nat. Commun..

[67] Walton Z.E., Patel C.H., Brooks R.C., Yu Y., Ibrahim-Hashim A., Riddle M., Porcu A., Jiang T., Ecker B.L., Tameire F. (2018). Acid Suspends the Circadian Clock in Hypoxia through Inhibition of mTOR. Cell.

[68] Abe M., Herzog E., Yamazaki S., Straume M., Tei H., Sakaki Y., Menaker M., Block G.D. (2002). Circadian Rhythms in Isolated Brain Regions. J. Neurosci..

[69] Ramanathan C., Stowie A., Smale L., Nunez A.A. (2010). Phase preference for the display of activity is associated with the phase of extra-suprachiasmatic nucleus oscillators within and between species. Neuroscience.

[70] Beaulé C., Granados-Fuentes D., Marpegan L., Herzog E.D. (2011). In vitro circadian rhythms: Imaging and electrophysiology. Essays Biochem..

[71] Prolo L.M., Takahashi J.S., Herzog E.D. (2005). Circadian Rhythm Generation and Entrainment in Astrocytes. J. Neurosci..

[72] Zhang S.L., Lahens N.F., Yue Z., Arnold D.M., Pakstis P.P., Schwarz J.E., Sehgal A. (2021). A circadian clock regulates efflux by the blood-brain barrier in mice and human cells. Nat. Commun..

[73] Keller M., Mazuch J., Abraham U., Eom G.D., Herzog E., Volk H.-D., Kramer A., Maier B. (2009). A circadian clock in macrophages controls inflammatory immune responses. Proc. Natl. Acad. Sci. USA.

[74] Slat E.A., Sponagel J., Marpegan L., Simon T., Kfoury N., Kim A., Binz A., Herzog E.D., Rubin J.B. (2017). Cell-intrinsic, Bmal1-dependent Circadian Regulation of Temozolomide Sensitivity in Glioblastoma. J. Biol. Rhythm..

[75] Zhanfeng N., Yanhui L., Zhou F., Shaocai H., Guangxing L., Hechun X. (2015). Circadian genes Per1 and Per2 increase radiosensitivity of glioma in vivo. Oncotarget.

[76] Shuboni-Mulligan D., Dahut M., Young D., Gilbert M., Armstrong T. (2019). Rdna-04. Circadian rhythms and radiation chronotherapy in glioblastoma cell lines and central nervous system cell controls. Neuro Oncol..

[77] Fuhr L., Abreu M., Carbone A., El-Athman R., Bianchi F., Laukkanen M.O., Mazzoccoli G., Relógio A. (2019). The Interplay between Colon Cancer Cells and Tumour-Associated Stromal Cells Impacts the Biological Clock and Enhances Malignant Phenotypes. Cancers.

[78] Masri S., Papagiannakopoulos T., Kinouchi K., Liu Y., Cervantes M., Baldi P., Jacks T., Sassone-Corsi P. (2016). Lung Adenocarcinoma Distally Rewires Hepatic Circadian Homeostasis. Cell.

[79] Grabowski M.M., Sankey E.W., Ryan K.J., Chongsathidkiet P., Lorrey S.J., Wilkinson D.S., Fecci P.E. (2021). Immune suppression in gliomas. J. Neuro Oncol..

[80] Chen P., Hsu W.-H., Chang A., Tan Z., Lan Z., Zhou A., Spring D.J., Lang F.F., Wang Y.A., Depinho R.A. (2020). Circadian Regulator CLOCK Recruits Immune-Suppressive Microglia into the GBM Tumor Microenvironment. Cancer Discov..

[81] Colaprico A., Silva T.C., Olsen C., Garofano L., Cava C., Garolini D., Sabedot T.S., Malta T.M., Pagnotta S.M., Castiglioni I. (2016). TCGAbiolinks: An R/Bioconductor package for integrative analysis of TCGA data. Nucleic Acids Res..

[82] Mounir M., Lucchetta M., Silva T.C., Olsen C., Bontempi G., Chen X., Noushmehr H., Colaprico A., Papaleo E. (2019). New functionalities in the TCGAbiolinks package for the study and integration of cancer data from GDC and GTEx. PLoS Comput. Biol..

[83] Silva T.C., Colaprico A., Olsen C., D’Angelo F., Bontempi G., Ceccarelli M., Noushmehr H. (2016). TCGA Workflow: Analyze cancer genomics and epigenomics data using Bioconductor packages. F1000Research.

[84] Díez-Villanueva A., Mallona I., Peinado M.A. (2015). Wanderer, an interactive viewer to explore DNA methylation and gene expression data in human cancer. Epigenetics Chromatin.

[85] Liberzon A., Birger C., Thorvaldsdóttir H., Ghandi M., Mesirov J.P., Tamayo P. (2015). The Molecular Signatures Database (MSigDB) hallmark gene set collection. Cell Syst..

[86] Subramanian A., Tamayo P., Mootha V.K., Mukherjee S., Ebert B.L., Gillette M.A., Paulovich A., Pomeroy S.L., Golub T.R., Lander E.S. (2005). Gene set enrichment analysis: A knowledge-based approach for interpreting genome-wide expression profiles. Proc. Natl. Acad. Sci. USA.

[87] Wilkerson M.D., Hayes D.N. (2010). ConsensusClusterPlus: A class discovery tool with confidence assessments and item tracking. Bioinformatics.

[88] Gu Z., Eils R., Schlesner M. (2016). Complex heatmaps reveal patterns and correlations in multidimensional genomic data. Bioinformatics.

[89] Huang D.W., Sherman B.T., Lempicki R.A. (2009). Systematic and integrative analysis of large gene lists using DAVID Bioinformatics Resources. Nat. Protoc..

[90] Huang D.W., Sherman B.T., Lempicki R.A. (2009). Bioinformatics enrichment tools: Paths toward the comprehensive functional analysis of large gene lists. Nucleic Acids Res..

[91] Ritchie M.E., Phipson B., Wu D., Hu Y., Law C.W., Shi W., Smyth G.K. (2015). limma powers differential expression analyses for RNA-sequencing and microarray studies. Nucleic Acids Res..

