# Supplementary material for: Association of Circadian Clock Gene Expression with Glioma Tumor Microenvironment and Patient Survival
Source: Cancers (Basel). 2021 Jun 2;13(11):2756. doi: 10.3390/cancers13112756 (PMC8199552; doi:10.3390/cancers13112756)
Supplement: Supplementary file 1 [file cancers-13-02756-s001.zip › cancers-1242428-supplementary.pdf]

cancers

MDPI

Supplementary Materials

Association of Circadian Clock Gene Expression with Glioma Tumor Microenvironment and Patient Survival

Julianie De La Cruz Minyety, Dorela D. Shuboni-Mulligan, Nicole Briceno, Demarrius Young Jr., Mark R. Gilbert, Orieta Celiku and Terri S. Armstrong

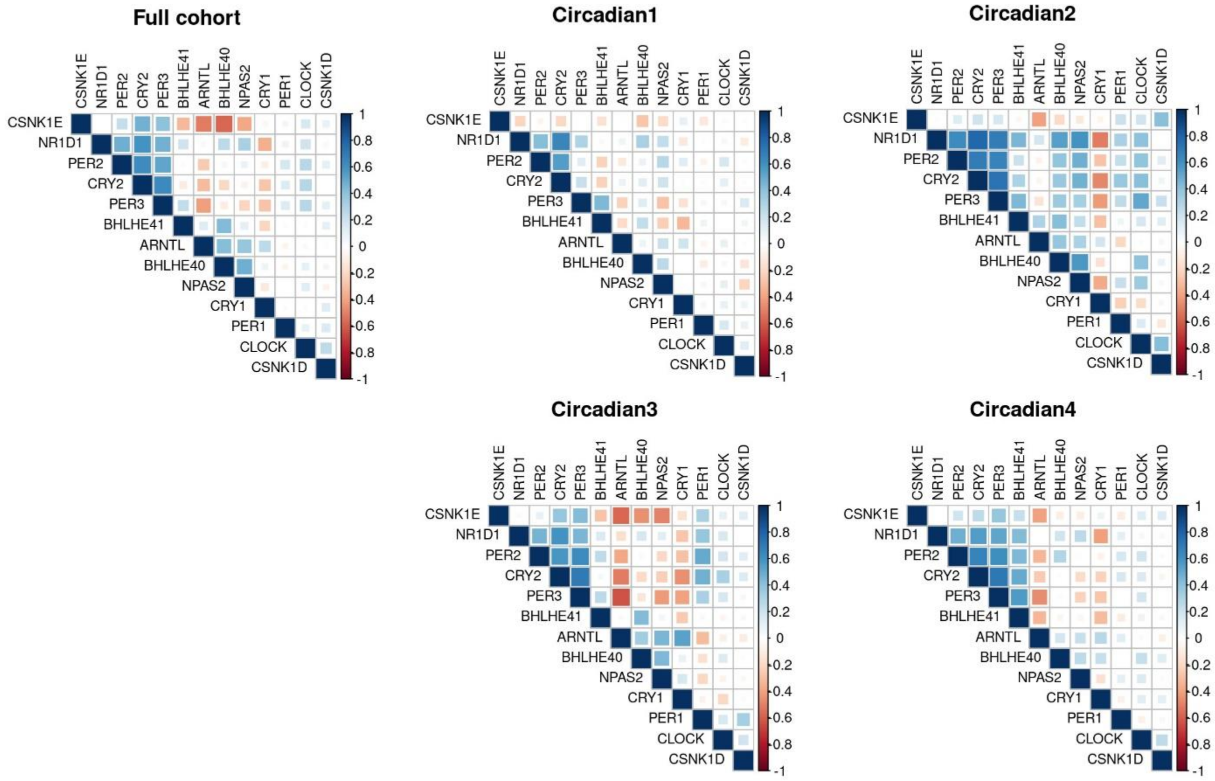

Figure S1. Graphs demonstrating the coordinated patterns of expression of clock genes in the TCGA data and unsupervised consensus clusters.

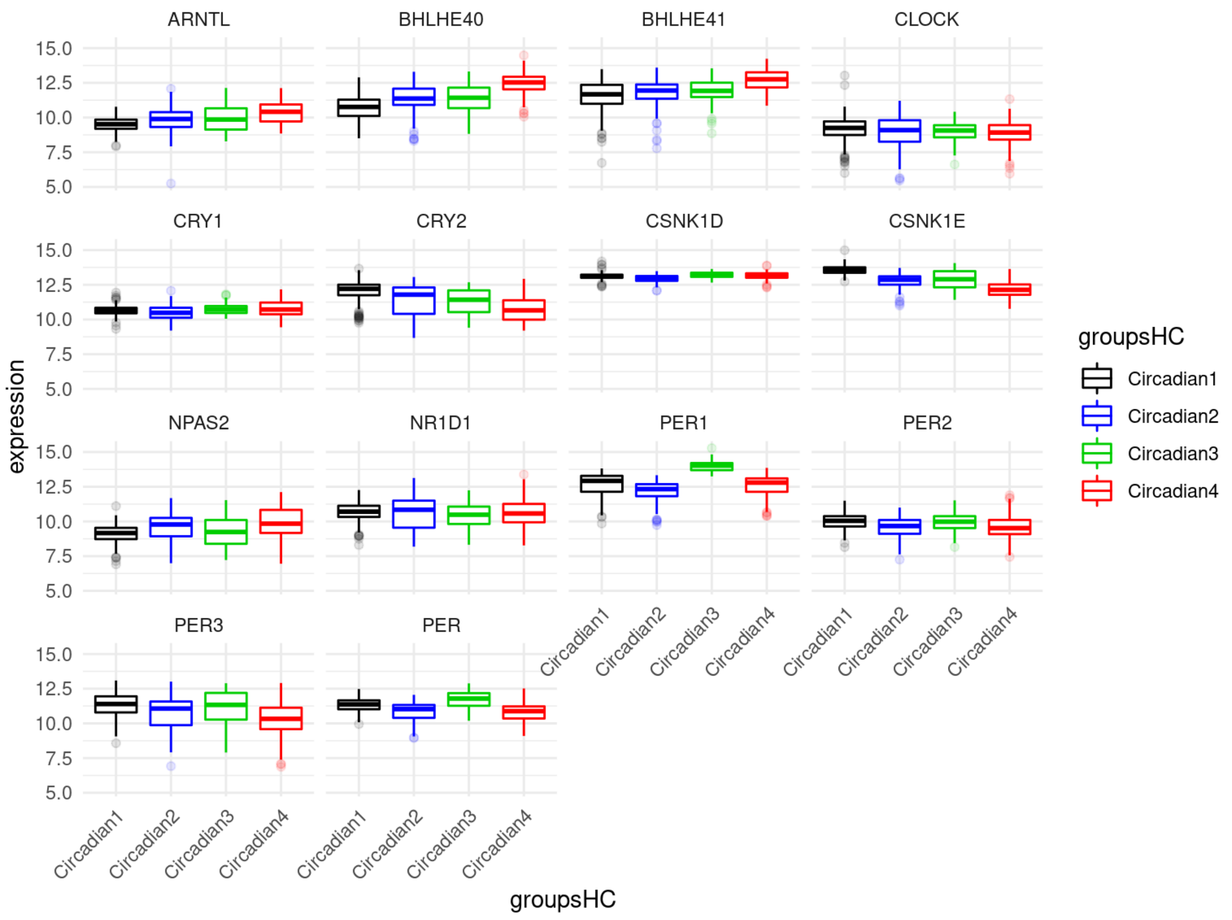

Figure S2. Clock gene expression in the Unsupervised clustering analysis.

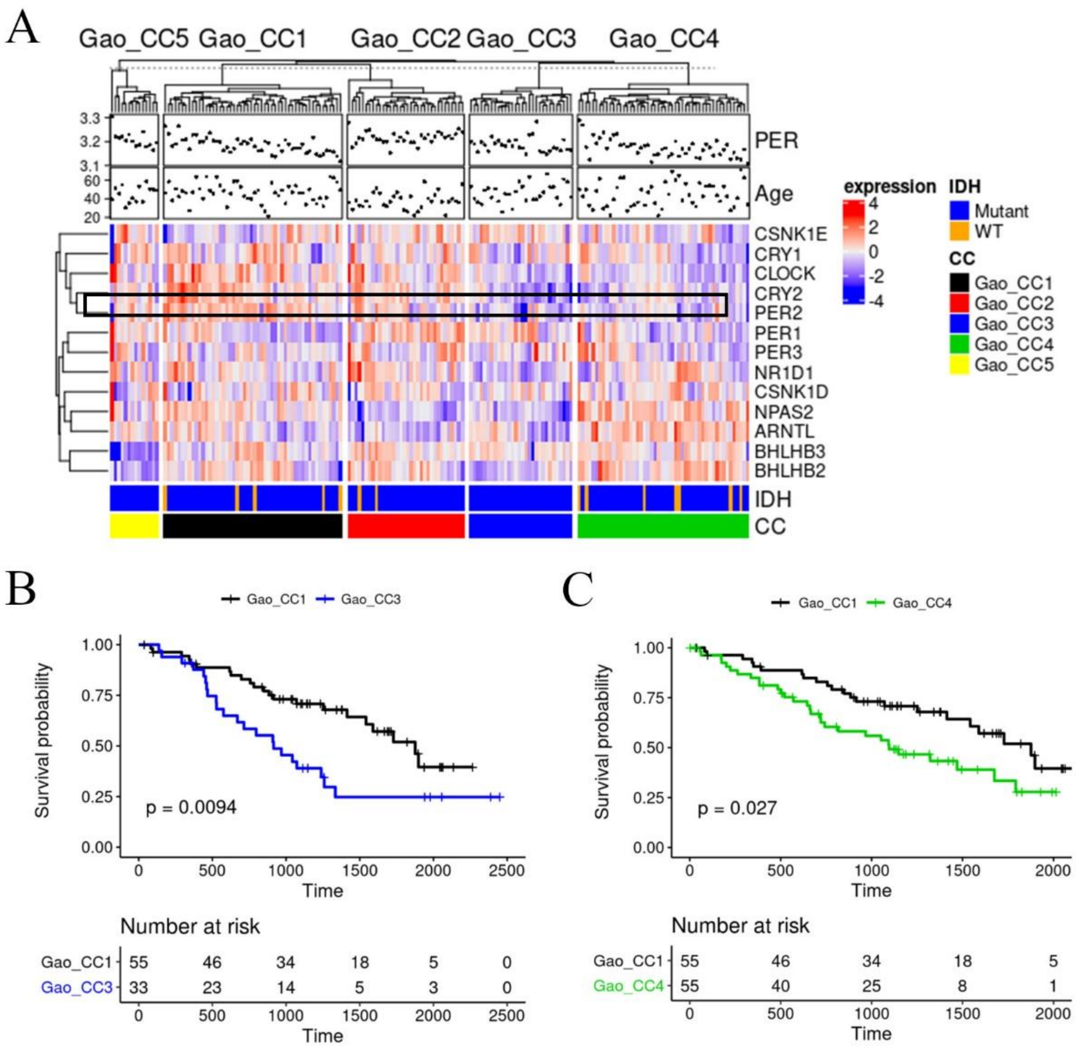

Figure S3. Prognostic relevance of PER in unsupervised clusters for LGG dataset.

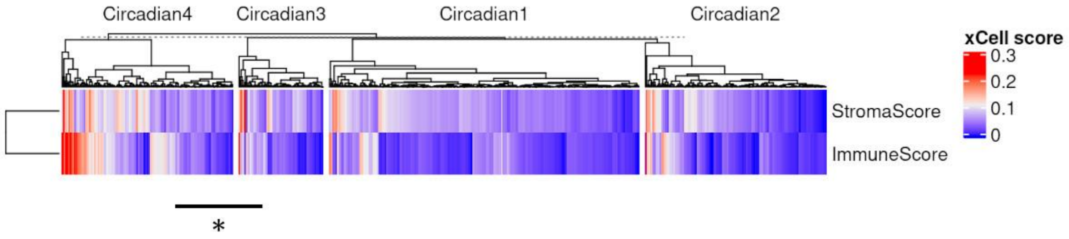

Figure S4. Contribution of different cell types in bulk tumor derived through a transcriptomic deconvolution algorithm.

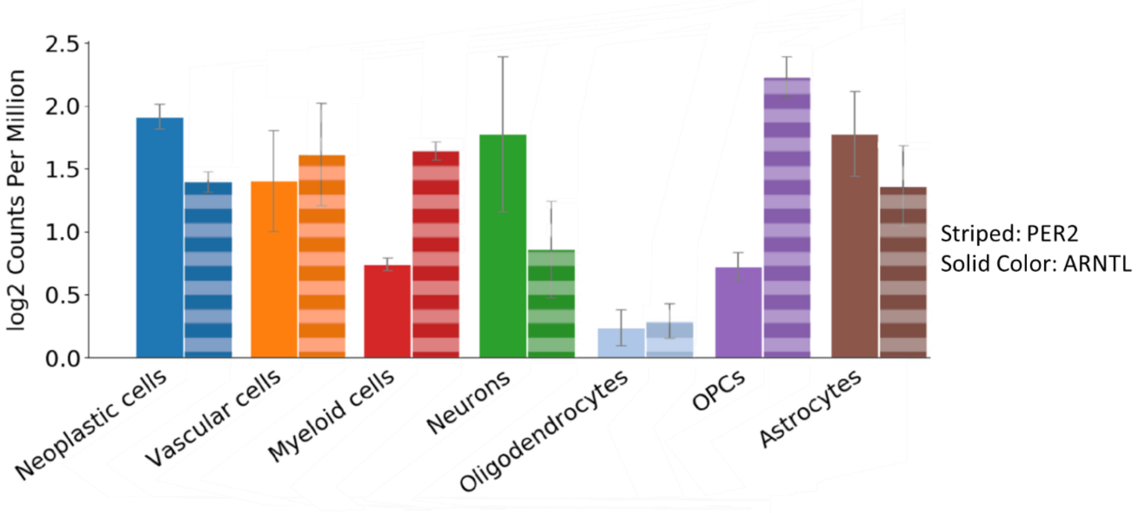

Figure S5. Differences in PER2 and ARNTL expression patterns between different cells of the tumor microenvironment.

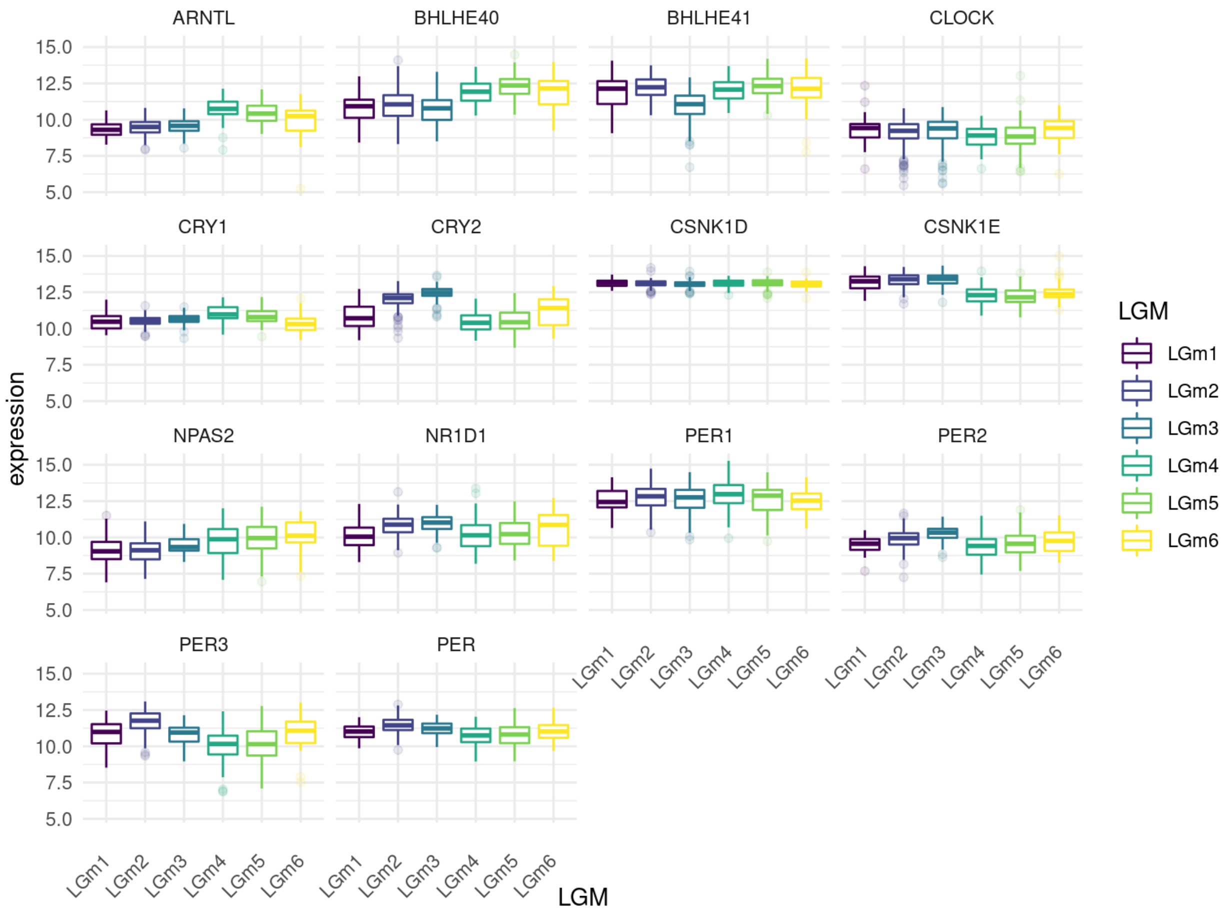

Figure S6. Clock gene expression in the subgroups separated by methylation status.

Table S1. Differences in clock gene expression between high and low PER expressing circadian clusters with predominantly IDH mutant or wild-type.

**Circadian2 vs Circadian1**					
**gene**	**logFC**	**logCPM**	**LR**	**PValue**	**FDR**
**ARNTL**	**0.5099157**	**4.36970964.5409047**		**0.00000000.0000000**	
**BHLHE40**	**0.6679979**	**5.78967669.4056474**		**0.00000000.0000000**	
**BHLHE41**	0.1215525	6.5048872.0096113		0.15630540.1934536	
**CLOCK**	-0.07549723.9439700.6899571			0.40617900.4585456	
**CRY1**	-0.06993245.2135172.5647545			0.10926950.1395630	
**CRY2**	**-0.48407696.62015535.6831852**			**0.00000000.0000000**	
**CSNK1D**	**-0.16271057.63195841.1579738**			**0.00000000.0000000**	
**CSNK1E**	**-0.74571537.924245358.51435130.00000000.0000000**				
**NPAS2**	**0.6526307**	**4.11445176.4362739**		**0.00000000.0000000**	
**NR1D1**	0.1313123	5.4331082.6794321		0.10165200.1305154	
**PER1**	**-0.52350857.24654552.0496282**			**0.00000000.0000000**	
**PER2**	**-0.39771074.54651643.1780577**			**0.00000000.0000000**	
**PER3**	**-0.43930305.95998824.5644610**			**0.00000070.0000018**	
**Circadian4 vs Circadian3**					
**gene**	logFC	logCPM	LR	PValue	FDR
**ARNTL**	0.2986951	5.0003976.9924615		0.00818540.0188575	
**BHLHE40**	**1.0120603**	**6.98270666.7986798**		**0.00000000.0000000**	
**BHLHE41**	**0.7587220**	**7.21510046.0548514**		**0.00000000.0000000**	
**CLOCK**	-0.04067913.7042230.1349282			0.71337610.7818568	
**CRY1**	0.0928333	5.4390471.3343710		0.24802910.3373284	
**CRY2**	**-0.57610735.76364522.8909132**			**0.00000170.0000122**	
**CSNK1D**	-0.03150967.7583610.6148198			0.43297880.5304049	
**CSNK1E**	**-0.79490897.11890094.6417929**			**0.00000000.0000000**	
**NPAS2**	**0.6088404**	**4.66425017.8250353**		**0.00002420.0001207**	
**NR1D1**	**0.3253224**	**5.4129506.1109049**		**0.01343500.0289239**	
**PER1**	**-1.29913807.870965211.71432270.00000000.0000000**				
**PER2**	**-0.25103324.5195525.1130381**			**0.02374670.0471488**	
**PER3**	**-0.68638625.67600819.5286806**			**0.00000990.0000556**	

Table S2. Kaplan-Meier analysis of overall survival between unsupervised consensus clusters.

	**Circadian1**	**Circadian4**	**Circadian2**
**Circadian4**	2×10^{-16}	-	-
**Circadian2**	4.9×10^{-7}	1.1×10^{-6}	-
**Circadian3**	1.3×10^{-6}	6.3×10^{-4}	0.7800

Table S3. Summary of full Cox model of the 13 core clock gene expression and reduced model of significant clock genes.

**Full model**					
	**coef**	**exp(coef)**	**se(coef)**	**z**	**Pr(>|z|)**
**IDHWT**	**1.355**	**3.877**	**0.307**	**4.407**	**<0.001**
**age**	**0.046**	**1.047**	**0.007**	**6.255**	**<0.001**
sexmale	-0.182	0.833	0.161	-1.133	0.257
ARNTL	0.157	1.169	0.115	1.366	0.172
BHLHE40	0.131	1.140	0.105	1.251	0.211
BHLHE41	0.027	1.027	0.108	0.251	0.802
CLOCK	0.097	1.102	0.104	0.937	0.349
CRY1	-0.171	0.842	0.185	-0.927	0.354
CRY2	-0.194	0.824	0.146	-1.328	0.184
**CSNK1D**	**0.862**	**2.368**	**0.306**	**2.817**	**0.005**
CSNK1E	0.028	1.028	0.168	0.164	0.869
NPAS2	-0.114	0.892	0.099	-1.154	0.248
NR1D1	0.117	1.125	0.135	0.872	0.383
PER1	0.093	1.097	0.086	1.081	0.280
**PER2**	**-0.425**	**0.654**	**0.148**	**-2.881**	**0.004**
PER3	-0.080	0.923	0.106	-0.751	0.452
**Reduced model**					
	**coef**	**exp(coef)**	**se(coef)**	**z**	**Pr(>|z|)**
**IDHWT**	1.671	5.318	0.214	7.821	**<0.001**
**age**	0.045	1.046	0.006	6.925	**<0.001**
sexmale	-0.130	0.878	0.157	-0.829	0.407
**CSNK1D**	0.942	2.565	0.299	3.151	**0.002**
**PER2**	-0.498	0.608	0.111	-4.491	**<0.001**

Table S4. Tumor composition analysis by deconvolution.

**A) Immune Score**				
**Comparison**	**Diff**	**lwr**	**upr**	**padj**
**Circadian4-Circadian1**	**0.0513**	**0.0391**	**0.0634**	**0.0000**
Circadian2-Circadian1	0.0024	-0.0096	0.0144	0.9550
Circadian3-Circadian1	0.0083	-0.0073	0.0240	0.5175
**Circadian2-Circadian4**	**-0.0489**	**-0.0625**	**-0.0352**	**0.0000**
**Circadian3-Circadian4**	**-0.0429**	**-0.0599**	**-0.0260**	**0.0000**
Circadian3-Circadian2	0.0059	-0.0109	0.0228	0.7996
**B) Stroma Score**				
**Comparison**	**Diff**	**lwr**	**upr**	**padj**
**Circadian4-Circadian1**	**0.0244**	**0.0144**	**0.0344**	**0.0000**
Circadian2-Circadian1	-0.0061	-0.0159	0.0038	0.3875
**Circadian3-Circadian1**	**0.0141**	**0.0013**	**0.0270**	**0.0250**
**Circadian2-Circadian4**	**-0.0305**	**-0.0417**	**-0.0193**	**0.0000**
Circadian3-Circadian4	-0.0103	-0.0242	0.0037	0.2303
**Circadian3-Circadian2**	**0.0202**	**0.0064**	**0.0340**	**0.0011**
